# Supplementary material for: IgM Antiphospholipid Antibodies in Antiphospholipid Syndrome: Prevalence, Clinical Associations, and Diagnostic Implications—A Scoping Review
Source: J Clin Med. 2025 Oct 11;14(20):7164. doi: 10.3390/jcm14207164 (PMC12565476; doi:10.3390/jcm14207164)
Supplement: Supplementary file 1 [file jcm-14-07164-s001.zip › IgM Review_Supplementary Data 2_Search queries.pdf]

SUPPLEMENTARY DATA 2

IgM Antiphospholipid Antibodies in Antiphospholipid Syndrome: Prevalence, Clinical Associations, and Diagnostic Implications— A Scoping Review

Monika Očková MSc[a], Ariadna Anunciación-Llunell MSc[a], Catalina Andrada MD[a], Enrique Esteve-Valverde MD[b], Francesc Miró-Mur PhD[a]*, Jaume Alijotas-Reig MD, PhD [a,c,d]*

Affiliations:

[a] Systemic Autoimmune Diseases Research Unit, Vall d'Hebron Institut de Recerca (VHIR). Passeig Vall d'Hebron 119-129. Barcelona 08035. Spain.

[b] Department of Internal Medicine, Hospital Sant Camil, Consorci Sanitari del Garraf, Barcelona 08810. Spain.

[c] Department of Internal Medicine, Hospital Universitari Vall d'Hebron (HUVH). Passeig Vall d'Hebron 119-129. Barcelona 08035. Spain.

[d] Department of Medicine, Universitat Autònoma de Barcelona (UAB). Vall d'Hebron Hospital Campus. Passeig Vall d'Hebron 119-129. Barcelona 08035. Spain.

***Corresponding authors**:

Francesc Miró-Mur, PhD

Systemic Autoimmune Diseases Research Unit, Vall d'Hebron Institut de Recerca (VHIR). Passeig Vall d'Hebron 119-129. 08035 Barcelona. Spain.

Phone: +34 93 737 2424; e-mail: *francesc.miro@vhir.org*

ORCID: *https://orcid.org/0000-0003-3936-2693*

Jaume Alijotas-Reig M.D, PhD, MSc

Department of Medicine, Universitat Autònoma de Barcelona (UAB). Vall d'Hebron Hospital Campus. Passeig Vall d'Hebron 119-129. Barcelona 08035. Spain.

Phone: +34 93 489 4194; Fax: +34 93 489 3039; e-mail: *jaime.alijotas@uab.cat*

ORCID: *www.ORCID.org/0000-0002-9212-3619*

1. Full search query (PubMed search 1) performed: June 16, 2025

APS, IgM, "anticardiolipin" OR "aβ2GPI" OR "beta-2 glycoprotein 1" OR "Beta-2-glycoprotein 1" OR "aPS/PT"

Specific records avoided (through filter selection): Adaptive Clinical Trial, Books and Documents, Case Reports, Classical Article, Clinical Conference, Clinical Study, Clinical Trial, Clinical Trial Protocol, Clinical Trial, Phase I, Clinical Trial, Phase II, Clinical Trial, Phase III, Clinical Trial, Phase IV, Clinical Trial, Veterinary, Collected Work, Comment, Comparative Study, Congress, Controlled Clinical Trial, Dataset, Directory, Editorial, Equivalence Trial, Evaluation Study, Guideline, Historical Article, Introductory Journal Article, Letter, Meta-Analysis, Multicenter Study, Network Meta-Analysis, Observational Study, Observational Study, Veterinary, Portrait, Practice Guideline, Pragmatic Clinical Trial, Preprint, Randomized Controlled Trial, Randomized Controlled Trial, Veterinary, Technical Report, Twin Study, Validation Study.

2. Full search query (PubMed search 2) performed: June 19, 2025

((("Antiphospholipid Syndrome"[MeSH Terms] OR "TAPS"[All Fields] OR "OAPS"[All Fields] OR "APS"[All Fields] OR "Antiphospholipid Syndrome"[All Fields]) AND ("Immunoglobulin M"[MeSH Terms] OR "IgM"[All Fields]) AND ("antibodies, antiphospholipid"[MeSH Terms] OR "aPL"[All Fields] OR "aPLs"[All Fields] OR "aB2GPI"[All Fields] OR "abeta2GPI"[All Fields] OR "aCL"[All Fields])) NOT "Systematic Review"[Title/Abstract]) AND (Prognosis/Narrow[filter])
